# Genomic analysis of aggressive ductal adenocarcinoma of the prostate

**DOI:** 10.1002/cam4.5573

**Published:** 2022-12-26

**Authors:** Hiroaki Kobayashi, Takeo Kosaka, Kohei Nakamura, Tokuhiro Kimura, Hiroshi Nishihara, Mototsugu Oya

**Affiliations:** ^1^ Department of Urology Keio University School of Medicine Tokyo Japan; ^2^ Department of Urology Saiseikai Yokohamashi Tobu Hospital Kanagawa Japan; ^3^ Genomics Unit, Keio Cancer Center Keio University School of Medicine Tokyo Japan; ^4^ Division of Diagnostic Pathology Saiseikai Yokohamashi Tobu Hospital Kanagawa Japan

**Keywords:** ductal adenocarcinoma, next generation sequencing, retinoblastoma transcriptional corepressor 1 (RB1), tumor suppressor protein p53 (TP53)

## Abstract

**Background:**

Genomic profile analysis using next‐generation sequencing can potentially elucidate the pathogenesis of rare cancers. Ductal adenocarcinoma, a rare subtype of prostate cancer, has an aggressive nature. This is the first study to analyze the genomic profile of ductal adenocarcinoma in an Asian population.

**Methods:**

We identified 12 patients newly diagnosed with ductal adenocarcinoma of the prostate at two hospitals, and nine patients (75.0%) had the pure type. Genomic assessment was performed using either the PleSSision testing platform or FoundationOne CDx.

**Results:**

At least one genomic alteration occurred in 11 patients (91.7%), and the most frequently mutated gene was tumor suppressor protein p53 (*TP53*), which was found in six cases (50.0%). Alterations characteristic of this cohort were found in four cases (33.3%) of retinoblastoma transcriptional corepressor 1 (*RB1*), which was only observed in the pure type. Compared to previous study results, the frequency of genetic alterations in the phosphoinositide 3‐kinase (PI3K) pathway (*n* = 3; 25.0%) and Wnt‐β‐catenin pathway (*n* = 5; 41.7%) was comparable, but no alterations in the DNA damage repair (DDR) pathway were observed. None of the patients presented high tumor mutation burden or microsatellite instability.

**Conclusions:**

We found that the Asian cohort with ductal adenocarcinoma had actionable alterations, and a high frequency of alterations in *TP53* and *RB1* reflected the aggressive nature of the tumor. Genetic analysis using next‐generation sequencing is expected to help elucidate the pathogenesis of ductal adenocarcinoma.

## Graphical abstract

This study is the first to analyze the genomic profile of ductal adenocarcinoma, that is a rare variant subtype of prostate cancer with an aggressive nature in an Asian population. We found that the Asian cohort with ductal adenocarcinoma was enriched for actionable alterations and a high frequency of alterations in TP53 and RB1 reflected its aggressive nature.

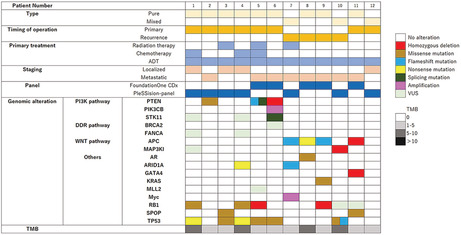

## 1 INTRODUCTION

Ductal adenocarcinoma is the most common subtype of prostate cancer; however, the pure type is rare, and represents only 0.1%–0.8% of all prostate cancers.^1^, ^2^ The mixed type with an acinar adenocarcinoma component is more common, representing 5.0%–12.3% of radical prostatectomy specimens.^3^, ^4^ Since existing reports have exclusively utilized population‐based studies and small institutional series owing to the cancer's rarity, the optimal management and genetic profile of ductal adenocarcinoma have not been established. In addition, ductal and acinar adenocarcinomas clonally originate from the same progenitor cell type, and are highly conserved (genome‐ and transcriptome‐wise) with the mixed type. These factors make it difficult to identify specific and reliable genomic biomarkers for ductal adenocarcinomas.^5^, ^6^, ^7^

To date, some studies have investigated the genetic profile of the ductal adenocarcinoma genotype, which has conserved upregulation of several pathways, including DNA damage repair (DDR), Wnt‐β‐catenin, and phosphoinositide 3‐kinase (PI3K) pathways, that drive the aggressive characteristics.^6^, ^7^, ^8^, ^9^

However, no study has established reliable evidence because of the small number of patients and limited geographic coverage. The discovery of novel prognostic biomarkers and identification of new pharmacotherapeutic targets for ductal adenocarcinoma are currently of research interest. In this study, for the first time, we aimed to summarize the genomic profiles of 12 Asian patients with prostate ductal adenocarcinoma.

## 2 METHODS

### 2.1 Patients

Approval for this study protocol was obtained from the ethics committees of Keio University Hospital (approval numbers 20160084 and 20180015) and Saiseikai Yokohamashi Tobu Hospital (approval number 20200102). Clinical and histological records from the two hospitals were searched for transurethral resection of the prostate (TURP) and prostate needle biopsy specimens with a ductal adenocarcinoma component, with available frozen tissues between January 2015 and December 2021. From these files, 12 patients with ductal components were identified. Archival formalin‐fixed paraffin‐embedded (FFPE) samples from surgical specimens (or 20 serial unstained slides) were collected and pre‐screened by board‐certified pathologists at Keio University to estimate the tumor content and storage duration of the specimens. Genomic testing was performed using either the PleSSision testing platform (PleSSision‐panel) or FoundationOne CDx (F1CDx).

### 2.2 Panel sequencing flow of primary tumors

#### 2.2.1 PleSSision‐panel

The detailed method of analysis using PleSSision‐panel has been described previously.^10^, ^11^ Genomic DNA was extracted and purified from pretreated primary tumor tissues using the QIAamp DNA Micro Kit (QIAGEN). Subsequently, DNA quality was checked based on DNA integrity number. Targeted amplicon next generation sequencing (NGS)‐based multiple gene assay involved 160 cancer‐related genes listed in Table S1. The sequencing data were entered into the original bioinformatic pipeline called GenomeJack (Mitsubishi Space Software Co., Ltd.) and analyzed within 3 days. We identified cancer‐specific alterations in somatic genes, including single‐nucleotide variations, copy number variations, and insertions/deletions. Tumor mutation burden (TMB) was determined using these findings.

#### 2.2.2 F1CDx

The F1CDx‐targeted NGS platform has been previously described and validated,^12^ and the methods are described briefly herein. Thin sections of FFPE tumor samples were sent to Foundation Medicine, Inc. After pathological review of the specimens, DNA was extracted and quantified before library construction. Libraries that passed quality control were hybridized and sequenced. Sequence data were analyzed using proprietary software developed by Foundation Medicine. F1CDx detected 324 genes (listed in Table S2), including all coding exons of 309 cancer‐related genes. Selected introns of 34 commonly rearranged genes and the coding exons of 21 genes were also included. In addition, F1CDx simultaneously profiled for TMB and microsatellite instability status.

## 3 RESULTS

### 3.1 Patient characteristics

Table 1 shows the clinicopathological parameters of the 12 patients. The median patient age at the time of diagnosis was 70 (range: 57–85) years and the median pretreatment PSA level was 5.6 (range: 1.2–129.0) ng/mL. Seven of the cases with PSA >4.0 had concurrent prostate needle biopsy with TURP. Nine patients (75.0%) had pure type cancer, while three patients (25.0%) had a mixed type acinar adenocarcinoma of the prostate according to the pathology reports. The majority of patients (*n* = 10; 83.3%) with tumor stage data had clinical stage T3 or higher disease. Patients with distant metastases at diagnosis had a particularly poor prognosis, with five patients (41.7%) dying of cancer during a median follow‐up of 25.0 months.

**TABLE 1:** Clinicopathological characteristics in 12 patients with ductal adenocarcinoma of the prostate

Case	Age	PSA	Needle biopsy	Gleason score	Type	cT stage	cN stage	Metastasis
1	84	2.04	−	4 + 4	Pure	2	0	−
2	67	6.41	+	4 + 4	Pure	3a	0	Lung
3	84	1.15	−	4 + 4	Pure	3a	0	−
4	85	1.34	−	4 + 4	Pure	3a	0	−
5	59	4.58	−	4 + 4	Pure	4	0	−
6	69	4.80	+	4 + 4	Pure	3a	1	−
7	85	4.87	−	4 + 4	Mixed	4	2	−
8	57	63.0	+	4 + 4	Pure	3a	0	Bone
9	71	129.0	+	4 + 4	Pure	4	2	Bone, Lung
10	69	14.16	+	4 + 4	Mixed	4	0	Bone
11	66	19.26	+	4 + 4	Pure	4	1	Lung
12	81	42.99	+	4 + 3	Mixed	2	0	−

### 3.2 Genomics of ductal adenocarcinoma of the prostate

**FIGURE 1:**
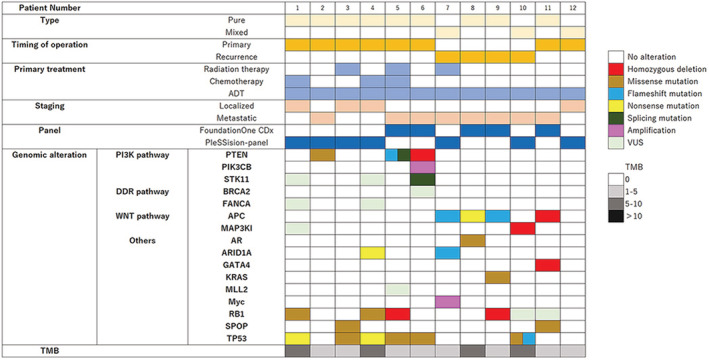
Landscape of genomic alterations in 12 patients with ductal adenocarcinoma of the prostate.

In our cohort, 11 patients (91.7%) had at least one genomic alteration (Figure 1). Frequently mutated genes were those that encoded tumor suppressor protein p53 (*TP53*) (*n* = 6; 50.0%) and retinoblastoma transcriptional corepressor 1 (*RB1*) (*n* = 4; 33.3%), as well as genes involved in the PI3K (*n* = 3; 25.0%) and Wnt‐β‐catenin (*n* = 5; 41.7%) pathways. However, no DDR pathway‐related changes were observed.

Genetic abnormalities in *TP53*, *RB1*, and phosphatase and tensin homolog deleted from chromosome 10 (*PTEN*) are frequently observed in human cancers and are often co‐mutated. In this cohort, *TP53* alterations were the most frequent, occurring in six cases (50.0%), and *RB1* alterations occurred in four cases (33.3%). There were three cases (Cases 1, 4, and 6) in which two of the three genes were mutated simultaneously and one case in which all three genes were mutated. All of these four cases were pure type with low PSA of less than 4.0 and no distant metastases at the time of diagnosis. However, disease progression was aggressive and prognosis was poor despite radiotherapy and drug treatment, and two patients died of cancer after a median follow‐up of 20.3 months.

Case 5 showed *PTEN* and *RB1* co‐loss and a *TP53* somatic mutation (p.R249G); such mutations are often found in patients with neuroendocrine prostate cancer. Case 5 presented the first instance of ductal adenocarcinoma mixed with acinar adenocarcinoma with a neuroendocrine phenotype, as previously reported.^13^ Among the three patients who had alterations in the PI3K pathways, all cases (Cases 2, 5, and 6) had *PTEN* alterations. Case 6 showed multiple alterations in *PTEN* loss, STK11 substitution at the splice site (c.374 + 1A > T), and phosphatidylinositol‐4,5‐bisphosphate 3‐kinase catalytic subunit β (PIK3CB) amplification in the PI3K pathway. In contrast, no genetic alterations in PIK3CA or Akt1 were observed.

In the Wnt‐β‐catenin pathway, adenomatous polyposis coli (APC) alterations occurred in four cases (33.3%) and mitogen‐activated protein kinase kinase kinase 1 (MAP3KI) alteration in one case (8.3%). However, there were no alterations in β‐catenin (CTNNB1) or WT1 expression in our cohort. Case 10 was the first case of ductal adenocarcinoma with a homozygous deletion of MAP3KI with a *TP53* somatic point mutation (p.Gly245 Ser), as previously reported.^14^

Two patients (16.7%) with speckle‐type BTB/POZ protein (SPOP) and AT‐rich interaction domain 1A (ARID1A) alterations were observed in this cohort. Although androgen receptor (AR) amplification is a relatively common genetic alteration in metastatic prostate cancer, some reports indicate that the alterations are less frequent in ductal adenocarcinomas (Table 2). No AR gene alterations were detected in any of the pre‐treatment specimens, but a point mutation (p.Q58L) was detected in one of the four specimens collected from the site of recurrence after the initial treatment. The microsatellite status could not be determined. No case was categorized to have a high TMB, as the TMB was less than six mutations per megabase of the genome coding area of DNA across all samples.

**TABLE 2:** The percentage of genomic alterations of ductal adenocarcinoma in this cohort compared with major studies in patients with ductal adenocarcinoma to date, sporadic localized prostate cancer (The TCGA data set), and advanced prostate cancer (The SU2C‐PCF International Prostate Cancer Dream Team discovery set)

Cancer		Ductal	Ductal	Ductal	Ductal	Ductal	Localized prostate cancer	Advanced prostate cancer
Location of study		Japan	Sweden^8^	USA^9^	USA^6^	USA and Canada^7^	USA^16^	USA^18^
Number of patients		12	11	11	10	51	333	150
Genes	PTEN	25.0%	–	36.4%	60.0%	15.7%	17.0%	40.7%
	PIK3CA	–	18.2%	9.1%	10.0%	17.6%	2.0%	5.3%
	PI3KCB	8.3%	–	–	–	–	0.5%	6.0%
	ATM	–	–	9.1%	10.0%	9.8%	6.0%	7.3%
	BRCA2	–	9.1%	–	–	17.6%	3.0%	13.3%
	FANCA	–	–	–	–	2.0%	–	–
	APC	33.3%	27.3%	–	10.0%	23.5%	–	8.7%
	CTNNB1	–	–	9.1%	30.0%	7.8%	2.0%	4.0%
	MAP3KI	8.3%	–	–	–	–	–	–
	AR	8.3%	–	9.1%	–	7.8%	–	62.7%
	ARID1A	16.7%	9.1%	–	–	–	1.2%	–
	FOXA1	–	18.2%	–	30.0%	33.3%	4.0%	12.0%
	GATA4	8.3%	–	–	–	–	–	–
	KRAS	8.3%	–	–	–	5.9%	–	–
	MLL2	8.3%	–	–	–	–	–	–
	Myc	8.3%	–	–	–	5.9%	7.0%	–
	RB1	33.3%	–	–	–	–	1.0%	9.3%
	SPOP	16.7%	9.1%	–	10.0%	11.8%	11.0%	8.0%
	TP53	50.0%	18.2%	45.4%	10.0%	17.6%	8.0%	53.3%

*Note*: The TCGA data set comprises 333 men with localized prostate cancer.^16^ The SU2C‐PCF International Prostate Cancer Dream Team discovery set comprises 150 men with metastatic castration‐resistant prostate cancer.^18^

## 4 DISCUSSION

Recent molecular profiling efforts have gradually identified distinct molecular subsets of acinar adenocarcinoma, from localized cancer to metastatic castration‐resistant prostate cancer (mCRPC).^15^, ^16^, ^17^, ^18^ Meanwhile, literature on genetic profiling of ductal adenocarcinoma has so far been limited to scattered reports from small cohorts. Table 2 shows the percentage of ductal adenocarcinoma alterations in this cohort and the differences from the genomic profiles of other major reports to date, the Cancer Genome Atlas (TCGA) primary localized prostate cancer study,^16^ and the Stand Up 2 Cancer‐Prostate Cancer Foundation (SU2C‐PCF) International Prostate Cancer Dream Team discovery set.^18^ When compared to primary acinar adenocarcinoma and mCRPC cohorts, we noted obvious differences in the mutational profile of ductal adenocarcinomas.

Ductal adenocarcinoma has various clinical features similar to other aggressive variants, such as neuroendocrine and androgen‐independent variant prostate cancers, including low PSA secretion and a preponderance of early visceral metastasis.^19^ These aggressive cancer subtypes are often associated with co‐loss of the potent tumor suppressor genes *TP53* and *RB1*, which is considered a poor prognostic factor.^20^, ^21^ In our study, *TP53* alterations were found in six cases (50.0%), a much higher rate than in other reports. Three of these cases had both *TP53* and *RB1* alterations. *RB1* alterations were found in four cases (33.3%), all in the pure type and none in the mixed type. However, the rates of *TP53* alterations in ductal adenocarcinoma was only 18% in the largest series (51 tumors).^7^ Cases with *RB1* alterations have not been reported by major case series or a recent study of 15 cases limited to mixed‐type ductal adenocarcinomas with a common clonal origin.^22^ The only case report of *PTEN* and *RB1* co‐loss, and *TP53* somatic mutation (p.R249G) has already been reported.^13^ These results of this study are very different from those reported in the United States and Western Europe, where *TP53* alterations are low and *RB1* alterations are absent, suggesting that *TP53* and *RB1* alterations may be more common in Asian patients with ductal adenocarcinoma and may have more aggressive characteristics.

The PI3K pathway contributes to basic cellular processes including protein synthesis, and cell growth and survival. It is also involved in the acquisition of therapeutic resistance in prostate cancer.^23^ Loss or alteration of *PTEN* or PIK3CA often results in hyperactive signaling of the PI3K‐Akt pathway, leading to an aggressive cancer phenotype. *PTEN* deficiency is important because it is associated with earlier intratumor divergence between the ductal and acinar adenocarcinoma components.^6^ Genomic characterization studies have demonstrated that 18%–70% of ductal adenocarcinomas have genetic alterations related to the PI3K pathway^6^, ^7^, ^8^, ^9^; this is consistent with the frequency of alterations in this study (33.3%). Although the reported incidence of *PTEN* alterations in East Asian patients with prostate cancer ranges from 10%–34%, which is lower than that in the United States and Western Europe (18%–40%),^24^, ^25^, ^26^ the results of this study suggest that East Asians patients with ductal adenocarcinoma may have *PTEN* alterations at a similar frequency.

The Wnt‐β‐catenin pathway is an essential driver of ductal morphogenesis in the developing prostate^27^ and plays roles in prostate cancer pathogenesis, cell proliferation, and drug resistance.^28^ Zhang et al. reported that the activation of the Wnt‐β‐catenin pathway and its interaction with AR play a major role in the acquisition of enzalutamide resistance.^29^ Alterations in the genes encoding APC and CTNNB1 are frequently mutated in not only lethal mCRPC but also ductal adenocarcinoma and upregulate Wnt‐β‐catenin pathway signaling^6^, ^7^, ^8^, ^9^, ^30^ Table 2 shows that APC alterations were found in four cases (33.3%), which is slightly more frequent than that in other studies on ductal adenocarcinoma (10%–27%)^6^, ^7^, ^8^, ^9^ and significantly more frequent than that in the SU2C‐PCF set (9%)^18^ in our cohort.

The DDR pathway is crucial in the context of genetic alterations in prostate cancer. In cases of acinar adenocarcinoma, these alterations are more frequent in metastatic cancer than in localized disease.^16^ Currently, targeted therapy with poly ADP‐ribose polymerase (PARP) inhibitors by genetic testing is covered by insurance in Japan, because PARP inhibitors have been shown to be effective in the presence of DDR alterations (especially in breast cancer susceptibility gene 2; BRCA2) in several cancers, including advanced prostate cancer.^31^, ^32^ Likewise, there are frequent alterations in the DDR pathway in patients with ductal adenocarcinoma, which have been reported to have higher rates than mCRPC of not only somatic mutations (20%–49%), but also autosomal‐dominant germline mutations (20%).^6^, ^7^, ^8^, ^9^, ^33^ However, no alterations in the DDR pathway were observed in the present study. Despite the small number of patients, this result may represent a genomic characteristic of Asian patients with ductal adenocarcinoma.

Some genes associated with the development and progression of prostate cancer, such as SPOP and forkhead box A1 (FOXA1), are known to have common alterations in ductal and acinar adenocarcinomas,^16^ and as shown in Table 2, SPOP alterations were found in 16.7% in this cohort. ARID1A encodes a protein that acts as a member of the SWI/SNF chromatin remodeling complex that promotes the expression of various genes. ARID1A alterations are found in cancers that are common among Asians, including ovarian clear cell carcinoma, gastric cancer, and biliary tract cancer.^34^, ^35^, ^36^ The frequency of ARID1A alterations in patients with ductal adenocarcinoma in this cohort may be characteristic of Asian populations.

The present study has several limitations. First, it was limited by its retrospective nature with a small number of patients owing to the rarity of ductal adenocarcinoma. In addition, the patient population was heterogeneous, with short median follow‐up. Patients who underwent radical prostatectomy were not included in this cohort. While we examined as many radical prostatectomy specimens as possible from the past 5 years, we could not find any specimens of ductal adenocarcinoma. This suggests that prostate ductal adenocarcinoma in Japan is less likely to be detected as a localized cancer because it follows a more aggressive course than that in Western countries. A larger multicenter study is thus warranted to confirm our findings.

In conclusion, this is the first study to summarize the genetic alterations in ductal adenocarcinoma of the prostate in an Asian cohort. The specific gene alteration in this cohort was *RB1*, which was mutated at a high frequency only in the pure type. Co‐mutation with *TP53* was also observed at a high frequency, supporting the aggressive nature of ductal adenocarcinoma. In addition, no genomic alterations were observed in the DDR pathway, whereas the PI3K and Wnt‐β‐catenin pathways, which are known to have high frequencies in other studies, showed similar gene alteration frequencies.

## AUTHOR CONTRIBUTIONS

**Hiroaki Kobayashi:** Conceptualization (equal); data curation (lead); formal analysis (equal); investigation (equal); methodology (equal); project administration (equal); writing – original draft (lead). **Takeo Kosaka:** Conceptualization (equal); data curation (supporting); formal analysis (equal); investigation (equal); methodology (equal); project administration (equal); writing – original draft (supporting). **Kohei Nakamura:** Conceptualization (supporting); data curation (supporting); formal analysis (equal); investigation (supporting); methodology (supporting); writing – original draft (supporting). **Tokuhiro Kimura:** Conceptualization (supporting); data curation (equal); formal analysis (supporting); investigation (supporting). **Hiroshi Nishihara:** Conceptualization (supporting); data curation (supporting); formal analysis (supporting); methodology (supporting); supervision (supporting). **Mototsugu Oya:** Conceptualization (supporting); methodology (supporting); project administration (supporting); supervision (lead); writing – original draft (supporting).

## FUNDING INFORMATION

This work was supported in part by a Grant‐in‐Aid for Scientific Research (#20H03817 to T.K, #21K19579 to T.K) from the Ministry of Education, Culture, Sports, Science, and Technology of Japan, and by a research grant to T. Kosaka from the Japan Urological Association.

## CONFLICT OF INTEREST

The authors have no conflict of interest to declare.

## ETHICAL APPROVAL

Approval for this study protocol was obtained from the ethics committees of Keio University Hospital (approval numbers 20160084 and 20180015) and Saiseikai Yokohamashi Tobu Hospital (approval number 20200102).

## INFORMED CONSENT

Consent for publication has been obtained from the patients in print. ‐ Registry and the Registration No. of the study/trial. N/A. ‐ Animal Studies. N/A.

## Supporting information


Table S1.

Table S2.

## Data Availability

Data are available on reasonable request. The data and materials used in the current study are available from the corresponding author on reasonable request.
