# Supplementary material for: Genomic analysis of aggressive ductal adenocarcinoma of the prostate
Source: Cancer Med. 2022 Dec 26;12(7):8445–51. doi: 10.1002/cam4.5573 (PMC10134333; doi:10.1002/cam4.5573)
Supplement: Supplementary file 2 — Table S2. [file CAM4-12-8445-s001.docx]

**Supplemental Table S2**. Total of 324 genes examined in the FoundationOne CDx test.

| ABL1 | ACVR1B | AKT1 | AKT2 | AKT3 | ALK | ALOX12B | AMER1 |
| --- | --- | --- | --- | --- | --- | --- | --- |
| APC | AR | ARAF | ARFRP1 | ARID1A | ASXL1 | ATM | ATR |
| ATRX | AURKA | AURKB | AXIN1 | AXL | BAP1 | BARD1 | BCL2 |
| BCL2L1 | BCL2L2 | BCL6 | BCOR | BCORL1 | BRAF | BRCA1 | BRCA2 |
| BRD4 | BRIP1 | BTG1 | BTG2 | BTK | C11orf30 | CALR | CARD11 |
| CASP8 | CBFB | CBL | CCND1 | CCND2 | CCND3 | CCNE1 | CD22 |
| CD274 | CD70 | CD79A | CD79B | CDC73 | CDH1 | CDK12 | CDK4 |
| CDK6 | CDK8 | CDKN1A | CDKN1B | CDKN2A | CDKN2B | CDKN2C | CEBPA |
| CHEK1 | CHEK2 | CIC | CREBBP | CRKL | CSF1R | CSF3R | CTCF |
| CTNNA1 | CTNNB1 | CUL3 | CUL4A | CXCR4 | CYP17A1 | DAXX | DDR1 |
| DDR2 | DIS3 | DNMT3A | DOT1L | EED | EGFR | EP300 | EPHA3 |
| EPHB1 | EPHB4 | ERBB2 | ERBB3 | ERBB4 | ERCC4 | ERG | ERRFI1 |
| ESR1 | EZH2 | FAM46C | FANCA | FANCC | FANCG | FANCL | FAS |
| FBXW7 | FGF10 | FGF12 | FGF14 | FGF19 | FGF23 | FGF3 | FGF4 |
| FGF6 | FGFR1 | FGFR2 | FGFR3 | FGFR4 | FH | FLCN | FLT1 |
| FLT3 | FOXL2 | FUBP1 | GABRA6 | GATA3 | GATA4 | GATA6 | GID4 |
| GNA11 | GNA13 | GNAQ | GNAS | GRM3 | GSK3B | H3F3A | HDAC1 |
| HGF | HNF1A | HRAS | HSD3B1 | ID3 | IDH1 | IDH2 | IFG1R |
| IKBKE | IKZF1 | INPP4B | IRF2 | IRF4 | IRS2 | JAK1 | JAK2 |
| JAK3 | JUN | KDM5A | KDM5C | KDM6A | KDR | KEAP1 | KEL |
| KIT | KLHL6 | KMT2A | KMT2D | KRAS | LTK | LYN | MAF |
| MAP2K1 | MAP2K2 | MAP2K4 | MAP3K1 | MAP3K13 | MAPK1 | MCL1 | MDM2 |
| MDM4 | MED12 | MEF2B | MEN1 | MERTK | MET | MITF | MKNK1 |
| MLH1 | MPL | MRE11A | MSH2 | MSH3 | MSH6 | MST1R | MTAP |
| MTOR | MUTYH | MYC | MYCL | MYCN | MYD88 | NBN | NF1 |
| NF2 | NFE2L2 | NFKBIA | NKX2-1 | NOTCH1 | NOTCH2 | NOTCH3 | NPM1 |
| NRAS | NT5C2 | NTRK1 | NTRK2 | NTRK3 | P2RY8 | PALB2 | PARK2 |
| PARP1 | PARP2 | PARP3 | PAX5 | PBRM1 | PDCD1 | PDCD1LG2 | PDGFRA |
| PDGFRB | PDK1 | PIK3C2B | PIK3C2G | PIK3CA | PIK3CB | PIK3R1 | PIM1 |
| PMS2 | POLD1 | POLE | PPARG | PPP2R1A | PPP2R2A | PRDM1 | PRKAR1A |
| PRKCI | PTCH1 | PTEN | PTPN11 | PTPRO | QKI | RAC1 | RAD21 |
| RAD51 | RAD51B | RAD51C | RAD51D | RAD52 | RAD54L | RAF1 | RARA |
| RB1 | RBM10 | REL | RET | RICTOR | RNF43 | ROS1 | RPTOR |
| SDHA | SDHB | SDHC | SDHD | SETD2 | SF3B1 | SGK1 | SMAD2 |
| SMAD4 | SMARCA4 | SMARCB1 | SMO | SNCAIP | SOCS1 | SOX2 | SOX9 |
| SPEN | SPOP | SRC | STAG2 | STAT3 | STK11 | SUFU | SYK |
| TBX3 | TEK | TET2 | TGFBR2 | TIPARP | TNFAIP3 | TNFRSF14 | TP53 |
| TSC1 | TSC2 | TYRO3 | U2AF1 | VEGFA | VHL | WHSC1 | WHSC1L1 |
| WT1 | XPO1 | XRCC2 | ZNF217 | ZNF703 |  |  |  |

| BCR | CD74 | ETV4 | ETV5 | ETV6 | EWSR1 | EZR | MYB |
| --- | --- | --- | --- | --- | --- | --- | --- |
| NUTM1 | RSPO2 | SDC4 | SLC34A2 | TERC | TERT | TMPRSS2 |  |
